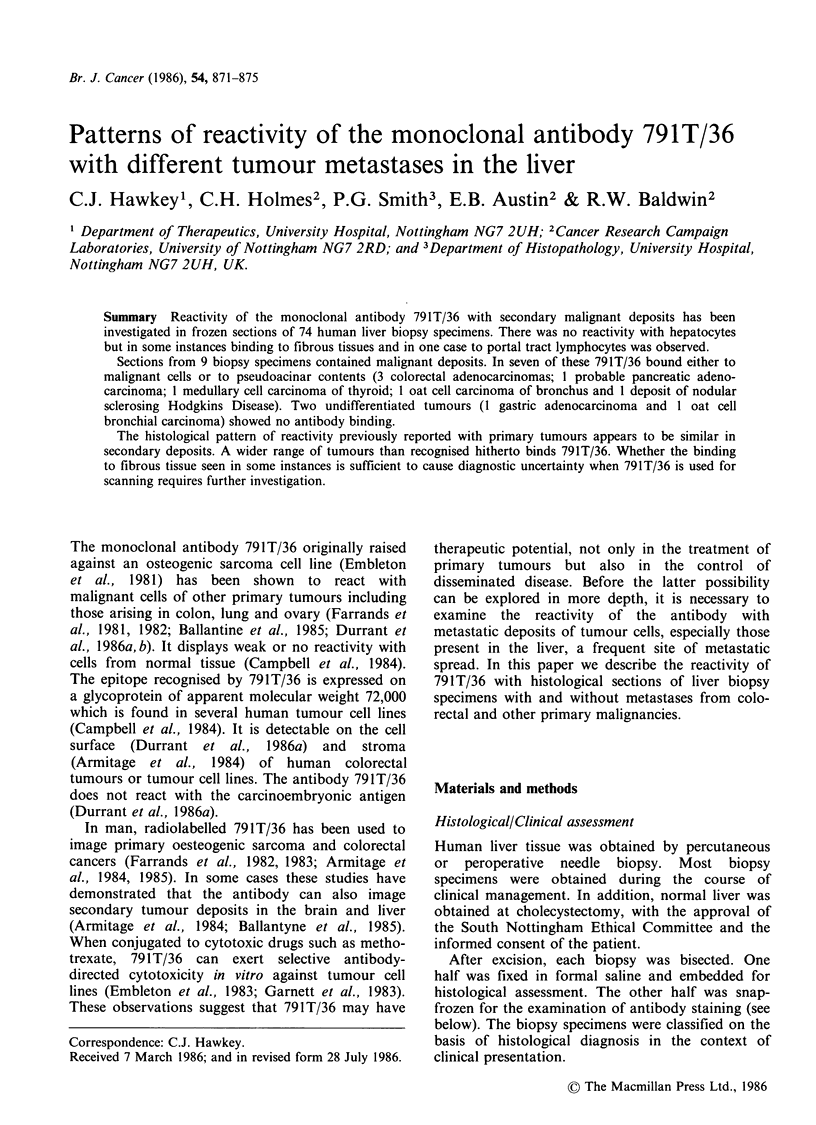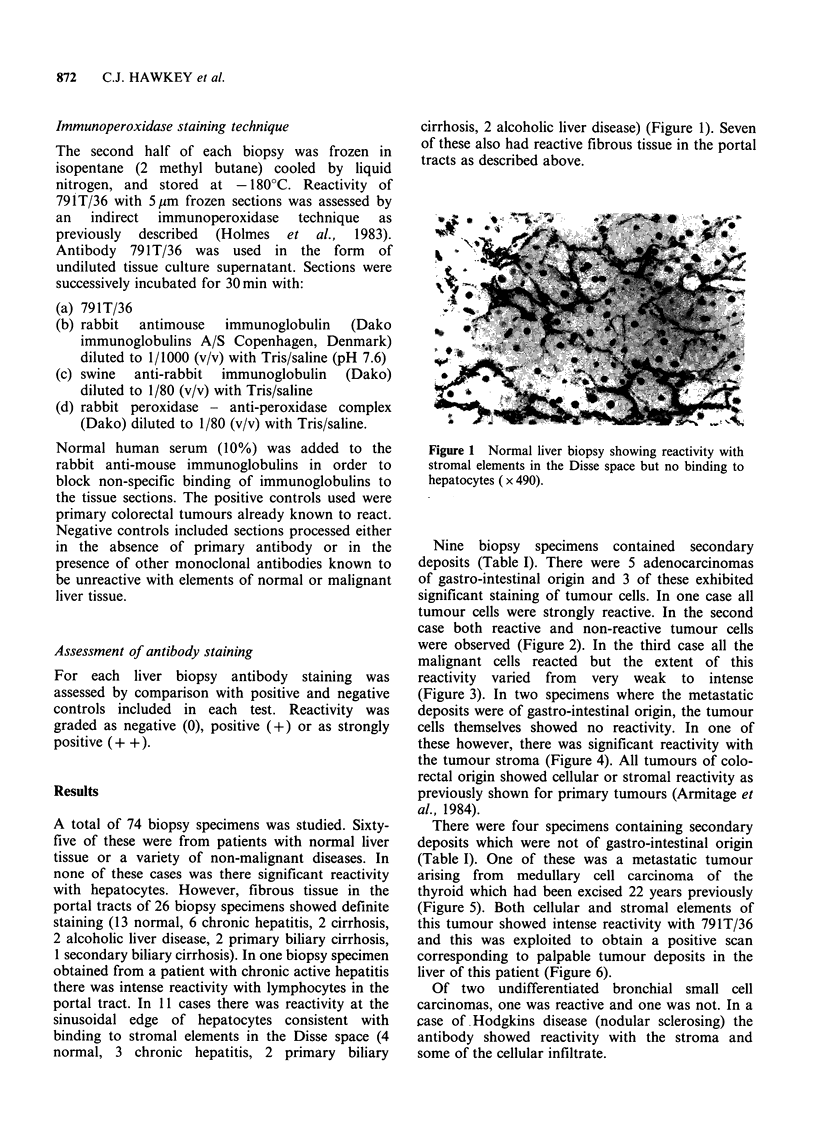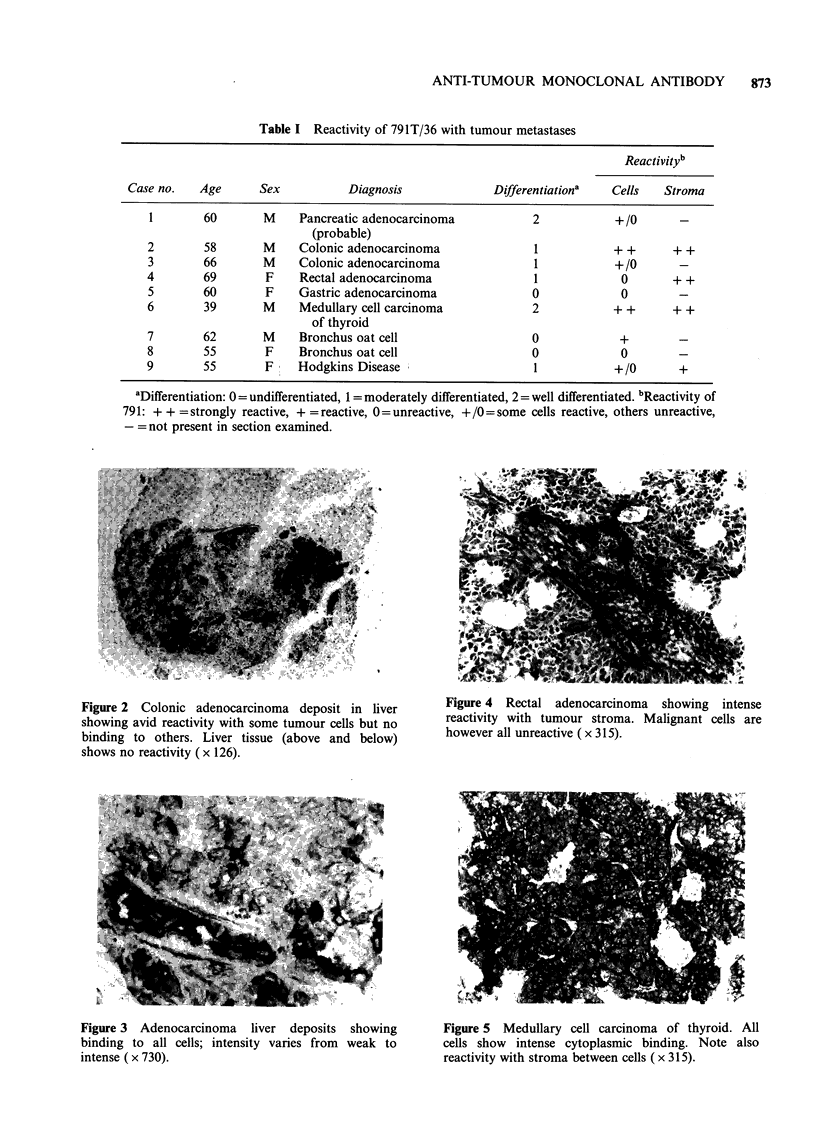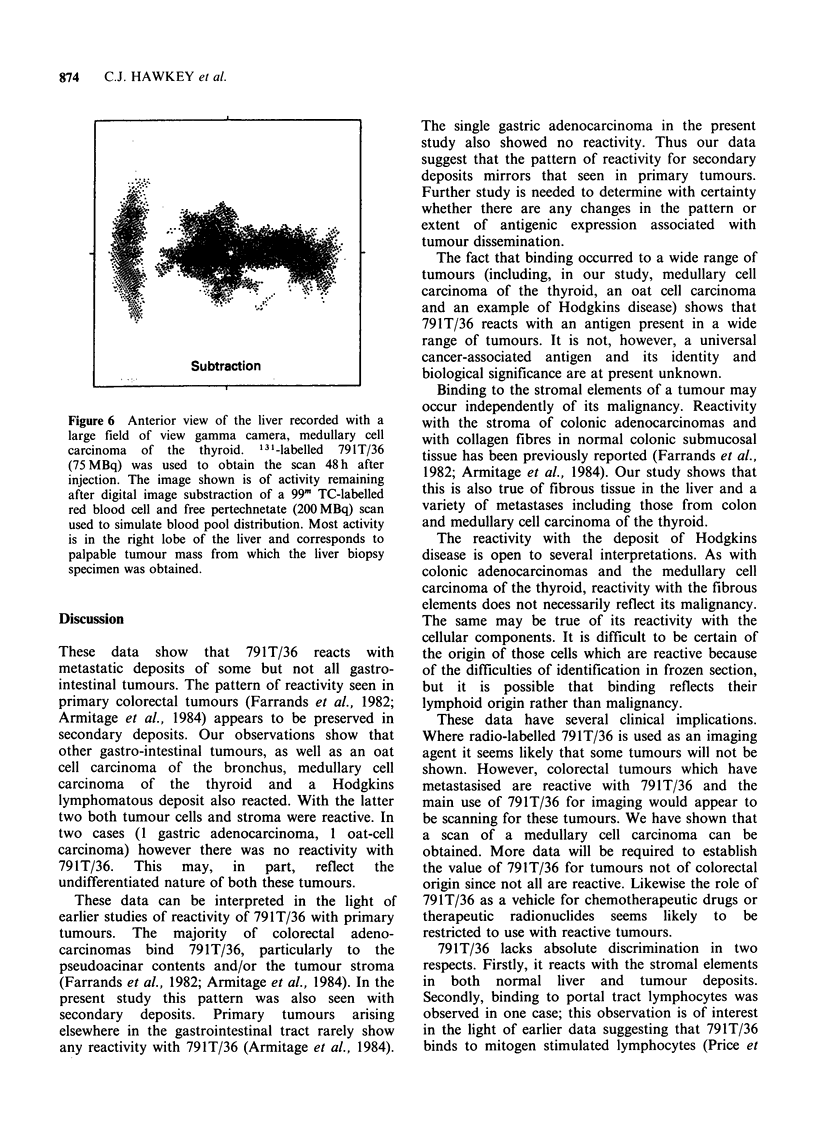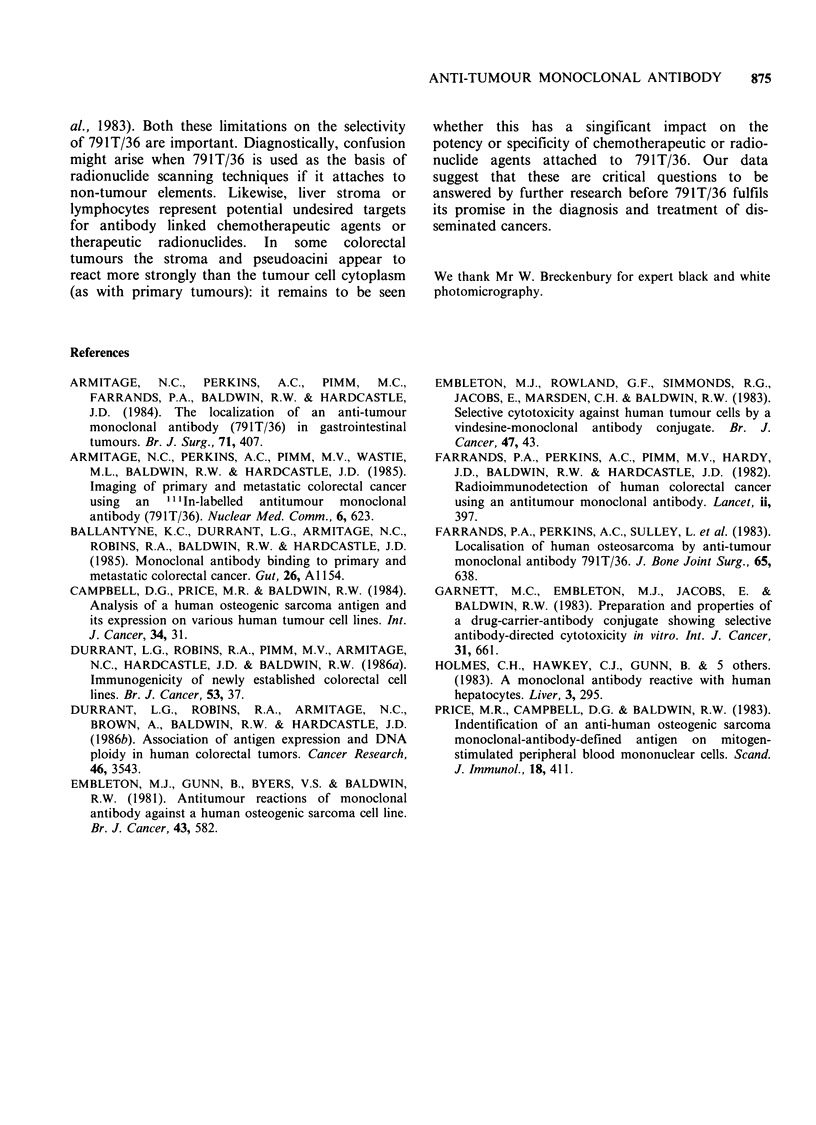# Patterns of reactivity of the monoclonal antibody 791T/36 with different tumour metastases in the liver.

**DOI:** 10.1038/bjc.1986.255

**Published:** 1986-12

**Authors:** C. J. Hawkey, C. H. Holmes, P. G. Smith, E. B. Austin, R. W. Baldwin

## Abstract

**Images:**


*Br. J. Cancer* (1986), **54**, 871–875

### Patterns of reactivity of the monoclonal antibody 791T/36 with different tumour metastases in the liver

C.J. Hawkey[1], C.H. Holmes[2], P.G. Smith[3], E.B. Austin[2] & R.W. Baldwin[2]

[1] *Department of Therapeutics, University Hospital, Nottingham NG7 2UH;* [2]*Cancer Research Campaign Laboratories, University of Nottingham NG7 2RD; and* [3]*Department of Histopathology, University Hospital, Nottingham NG7 2UH, UK.*

**Summary** Reactivity of the monoclonal antibody 791T/36 with secondary malignant deposits has been investigated in frozen sections of 74 human liver biopsy specimens. There was no reactivity with hepatocytes but in some instances binding to fibrous tissues and in one case to portal tract lymphocytes was observed.

Sections from 9 biopsy specimens contained malignant deposits. In seven of these 791T/36 bound either to malignant cells or to pseudoacinar contents (3 colorectal adenocarcinomas; 1 probable pancreatic adenocarcinoma; 1 medullary cell carcinoma of thyroid; 1 oat cell carcinoma of bronchus and 1 deposit of nodular sclerosing Hodgkins Disease). Two undifferentiated tumours (1 gastric adenocarcinoma and 1 oat cell bronchial carcinoma) showed no antibody binding.

The histological pattern of reactivity previously reported with primary tumours appears to be similar in secondary deposits. A wider range of tumours than recognised hitherto binds 791T/36. Whether the binding to fibrous tissue seen in some instances is sufficient to cause diagnostic uncertainty when 791T/36 is used for scanning requires further investigation.

The monoclonal antibody 791T/36 originally raised against an osteogenic sarcoma cell line (Embleton *et al.*, 1981) has been shown to react with malignant cells of other primary tumours including those arising in colon, lung and ovary (Farrands *et al.*, 1981, 1982; Ballantine *et al.*, 1985; Durrant *et al.*, 1986*a, b*). It displays weak or no reactivity with cells from normal tissue (Campbell *et al.*, 1984). The epitope recognised by 791T/36 is expressed on a glycoprotein of apparent molecular weight 72,000 which is found in several human tumour cell lines (Campbell *et al.*, 1984). It is detectable on the cell surface (Durrant *et al.*, 1986*a*) and stroma (Armitage *et al.*, 1984) of human colorectal tumours or tumour cell lines. The antibody 791T/36 does not react with the carcinoembryonic antigen (Durrant *et al.*, 1986*a*).

In man, radiolabelled 791T/36 has been used to image primary oesteogenic sarcoma and colorectal cancers (Farrands *et al.*, 1982, 1983; Armitage *et al.*, 1984, 1985). In some cases these studies have demonstrated that the antibody can also image secondary tumour deposits in the brain and liver (Armitage *et al.*, 1984; Ballantyne *et al.*, 1985). When conjugated to cytotoxic drugs such as methotrexate, 791T/36 can exert selective antibody-directed cytotoxicity *in vitro* against tumour cell lines (Embleton *et al.*, 1983; Garnett *et al.*, 1983). These observations suggest that 791T/36 may have therapeutic potential, not only in the treatment of primary tumours but also in the control of disseminated disease. Before the latter possibility can be explored in more depth, it is necessary to examine the reactivity of the antibody with metastatic deposits of tumour cells, especially those present in the liver, a frequent site of metastatic spread. In this paper we describe the reactivity of 791T/36 with histological sections of liver biopsy specimens with and without metastases from colorectal and other primary malignancies.

Correspondence: C.J. Hawkey.
Received 7 March 1986; and in revised form 28 July 1986.

### Materials and methods

### *Histological/Clinical assessment*

Human liver tissue was obtained by percutaneous or peroperative needle biopsy. Most biopsy specimens were obtained during the course of clinical management. In addition, normal liver was obtained at cholecystectomy, with the approval of the South Nottingham Ethical Committee and the informed consent of the patient.

After excision, each biopsy was bisected. One half was fixed in formal saline and embedded for histological assessment. The other half was snap-frozen for the examination of antibody staining (see below). The biopsy specimens were classified on the basis of histological diagnosis in the context of clinical presentation.

© The Macmillan Press Ltd., 1986

*Immunoperoxidase staining technique*

The second half of each biopsy was frozen in isopentane (2 methyl butane) cooled by liquid nitrogen, and stored at −180°C. Reactivity of 791T/36 with 5 μm frozen sections was assessed by an indirect immunoperoxidase technique as previously described (Holmes *et al.*, 1983). Antibody 791T/36 was used in the form of undiluted tissue culture supernatant. Sections were successively incubated for 30 min with:

(a) 791T/36
(b) rabbit antimouse immunoglobulin (Dako immunoglobulins A/S Copenhagen, Denmark) diluted to 1/1000 (v/v) with Tris/saline (pH 7.6)
(c) swine anti-rabbit immunoglobulin (Dako) diluted to 1/80 (v/v) with Tris/saline
(d) rabbit peroxidase – anti-peroxidase complex (Dako) diluted to 1/80 (v/v) with Tris/saline.

Normal human serum (10%) was added to the rabbit anti-mouse immunoglobulins in order to block non-specific binding of immunoglobulins to the tissue sections. The positive controls used were primary colorectal tumours already known to react. Negative controls included sections processed either in the absence of primary antibody or in the presence of other monoclonal antibodies known to be unreactive with elements of normal or malignant liver tissue.

*Assessment of antibody staining*

For each liver biopsy antibody staining was assessed by comparison with positive and negative controls included in each test. Reactivity was graded as negative (0), positive (+) or as strongly positive (+ +).

### Results

A total of 74 biopsy specimens was studied. Sixty-five of these were from patients with normal liver tissue or a variety of non-malignant diseases. In none of these cases was there significant reactivity with hepatocytes. However, fibrous tissue in the portal tracts of 26 biopsy specimens showed definite staining (13 normal, 6 chronic hepatitis, 2 cirrhosis, 2 alcoholic liver disease, 2 primary biliary cirrhosis, 1 secondary biliary cirrhosis). In one biopsy specimen obtained from a patient with chronic active hepatitis there was intense reactivity with lymphocytes in the portal tract. In 11 cases there was reactivity at the sinusoidal edge of hepatocytes consistent with binding to stromal elements in the Disse space (4 normal, 3 chronic hepatitis, 2 primary biliary cirrhosis, 2 alcoholic liver disease) (Figure 1). Seven of these also had reactive fibrous tissue in the portal tracts as described above.

**Figure 1** Normal liver biopsy showing reactivity with stromal elements in the Disse space but no binding to hepatocytes (×490).

Nine biopsy specimens contained secondary deposits (Table I). There were 5 adenocarcinomas of gastro-intestinal origin and 3 of these exhibited significant staining of tumour cells. In one case all tumour cells were strongly reactive. In the second case both reactive and non-reactive tumour cells were observed (Figure 2). In the third case all the malignant cells reacted but the extent of this reactivity varied from very weak to intense (Figure 3). In two specimens where the metastatic deposits were of gastro-intestinal origin, the tumour cells themselves showed no reactivity. In one of these however, there was significant reactivity with the tumour stroma (Figure 4). All tumours of colorectal origin showed cellular or stromal reactivity as previously shown for primary tumours (Armitage *et al.*, 1984).

There were four specimens containing secondary deposits which were not of gastro-intestinal origin (Table I). One of these was a metastatic tumour arising from medullary cell carcinoma of the thyroid which had been excised 22 years previously (Figure 5). Both cellular and stromal elements of this tumour showed intense reactivity with 791T/36 and this was exploited to obtain a positive scan corresponding to palpable tumour deposits in the liver of this patient (Figure 6).

Of two undifferentiated bronchial small cell carcinomas, one was reactive and one was not. In a case of Hodgkins disease (nodular sclerosing) the antibody showed reactivity with the stroma and some of the cellular infiltrate.

**Table I** Reactivity of 791T/36 with tumour metastases

| *Case no.* | *Age* | *Sex* | *Diagnosis* | *Differentiation*[a] | *Reactivity*[b] *Cells* | *Stroma* |
|---|---|---|---|---|---|---|
| 1 | 60 | M | Pancreatic adenocarcinoma (probable) | 2 | +/0 | – |
| 2 | 58 | M | Colonic adenocarcinoma | 1 | ++ | ++ |
| 3 | 66 | M | Colonic adenocarcinoma | 1 | +/0 | – |
| 4 | 69 | F | Rectal adenocarcinoma | 1 | 0 | ++ |
| 5 | 60 | F | Gastric adenocarcinoma | 0 | 0 | – |
| 6 | 39 | M | Medullary cell carcinoma of thyroid | 2 | ++ | ++ |
| 7 | 62 | M | Bronchus oat cell | 0 | + | – |
| 8 | 55 | F | Bronchus oat cell | 0 | 0 | – |
| 9 | 55 | F | Hodgkins Disease | 1 | +/0 | + |

[a]Differentiation: 0 = undifferentiated, 1 = moderately differentiated, 2 = well differentiated. [b]Reactivity of 791: ++ = strongly reactive, + = reactive, 0 = unreactive, +/0 = some cells reactive, others unreactive, – = not present in section examined.

**Figure 2** Colonic adenocarcinoma deposit in liver showing avid reactivity with some tumour cells but no binding to others. Liver tissue (above and below) shows no reactivity (×126).

**Figure 4** Rectal adenocarcinoma showing intense reactivity with tumour stroma. Malignant cells are however all unreactive (×315).

**Figure 3** Adenocarcinoma liver deposits showing binding to all cells; intensity varies from weak to intense (×730).

**Figure 5** Medullary cell carcinoma of thyroid. All cells show intense cytoplasmic binding. Note also reactivity with stroma between cells (×315).

**Figure 6** Anterior view of the liver recorded with a large field of view gamma camera, medullary cell carcinoma of the thyroid. $^{131}$-labelled 791T/36 (75 MBq) was used to obtain the scan 48 h after injection. The image shown is of activity remaining after digital image substraction of a $99^m$ TC-labelled red blood cell and free pertechnetate (200 MBq) scan used to simulate blood pool distribution. Most activity is in the right lobe of the liver and corresponds to palpable tumour mass from which the liver biopsy specimen was obtained.

### Discussion

These data show that 791T/36 reacts with metastatic deposits of some but not all gastro-intestinal tumours. The pattern of reactivity seen in primary colorectal tumours (Farrands *et al.*, 1982; Armitage *et al.*, 1984) appears to be preserved in secondary deposits. Our observations show that other gastro-intestinal tumours, as well as an oat cell carcinoma of the bronchus, medullary cell carcinoma of the thyroid and a Hodgkins lymphomatous deposit also reacted. With the latter two both tumour cells and stroma were reactive. In two cases (1 gastric adenocarcinoma, 1 oat-cell carcinoma) however there was no reactivity with 791T/36. This may, in part, reflect the undifferentiated nature of both these tumours.

These data can be interpreted in the light of earlier studies of reactivity of 791T/36 with primary tumours. The majority of colorectal adenocarcinomas bind 791T/36, particularly to the pseudoacinar contents and/or the tumour stroma (Farrands *et al.*, 1982; Armitage *et al.*, 1984). In the present study this pattern was also seen with secondary deposits. Primary tumours arising elsewhere in the gastrointestinal tract rarely show any reactivity with 791T/36 (Armitage *et al.*, 1984). The single gastric adenocarcinoma in the present study also showed no reactivity. Thus our data suggest that the pattern of reactivity for secondary deposits mirrors that seen in primary tumours. Further study is needed to determine with certainty whether there are any changes in the pattern or extent of antigenic expression associated with tumour dissemination.

The fact that binding occurred to a wide range of tumours (including, in our study, medullary cell carcinoma of the thyroid, an oat cell carcinoma and an example of Hodgkins disease) shows that 791T/36 reacts with an antigen present in a wide range of tumours. It is not, however, a universal cancer-associated antigen and its identity and biological significance are at present unknown.

Binding to the stromal elements of a tumour may occur independently of its malignancy. Reactivity with the stroma of colonic adenocarcinomas and with collagen fibres in normal colonic submucosal tissue has been previously reported (Farrands *et al.*, 1982; Armitage *et al.*, 1984). Our study shows that this is also true of fibrous tissue in the liver and a variety of metastases including those from colon and medullary cell carcinoma of the thyroid.

The reactivity with the deposit of Hodgkins disease is open to several interpretations. As with colonic adenocarcinomas and the medullary cell carcinoma of the thyroid, reactivity with the fibrous elements does not necessarily reflect its malignancy. The same may be true of its reactivity with the cellular components. It is difficult to be certain of the origin of those cells which are reactive because of the difficulties of identification in frozen section, but it is possible that binding reflects their lymphoid origin rather than malignancy.

These data have several clinical implications. Where radio-labelled 791T/36 is used as an imaging agent it seems likely that some tumours will not be shown. However, colorectal tumours which have metastasised are reactive with 791T/36 and the main use of 791T/36 for imaging would appear to be scanning for these tumours. We have shown that a scan of a medullary cell carcinoma can be obtained. More data will be required to establish the value of 791T/36 for tumours not of colorectal origin since not all are reactive. Likewise the role of 791T/36 as a vehicle for chemotherapeutic drugs or therapeutic radionuclides seems likely to be restricted to use with reactive tumours.

791T/36 lacks absolute discrimination in two respects. Firstly, it reacts with the stromal elements in both normal liver and tumour deposits. Secondly, binding to portal tract lymphocytes was observed in one case; this observation is of interest in the light of earlier data suggesting that 791T/36 binds to mitogen stimulated lymphocytes (Price *et*

*al.*, 1983). Both these limitations on the selectivity of 791T/36 are important. Diagnostically, confusion might arise when 791T/36 is used as the basis of radionuclide scanning techniques if it attaches to non-tumour elements. Likewise, liver stroma or lymphocytes represent potential undesired targets for antibody linked chemotherapeutic agents or therapeutic radionuclides. In some colorectal tumours the stroma and pseudoacini appear to react more strongly than the tumour cell cytoplasm (as with primary tumours): it remains to be seen whether this has a singificant impact on the potency or specificity of chemotherapeutic or radionuclide agents attached to 791T/36. Our data suggest that these are critical questions to be answered by further research before 791T/36 fulfils its promise in the diagnosis and treatment of disseminated cancers.

We thank Mr W. Breckenbury for expert black and white photomicrography.

**References**

ARMITAGE, N.C., PERKINS, A.C., PIMM, M.C., FARRANDS, P.A., BALDWIN, R.W. & HARDCASTLE, J.D. (1984). The localization of an anti-tumour monoclonal antibody (791T/36) in gastrointestinal tumours. *Br. J. Surg.*, **71**, 407.

ARMITAGE, N.C., PERKINS, A.C., PIMM, M.V., WASTIE, M.L., BALDWIN, R.W. & HARDCASTLE, J.D. (1985). Imaging of primary and metastatic colorectal cancer using an $^{111}$In-labelled antitumour monoclonal antibody (791T/36). *Nuclear Med. Comm.*, **6**, 623.

BALLANTYNE, K.C., DURRANT, L.G., ARMITAGE, N.C., ROBINS, R.A., BALDWIN, R.W. & HARDCASTLE, J.D. (1985). Monoclonal antibody binding to primary and metastatic colorectal cancer. *Gut*, **26**, A1154.

CAMPBELL, D.G., PRICE, M.R. & BALDWIN, R.W. (1984). Analysis of a human osteogenic sarcoma antigen and its expression on various human tumour cell lines. *Int. J. Cancer*, **34**, 31.

DURRANT, L.G., ROBINS, R.A., PIMM, M.V., ARMITAGE, N.C., HARDCASTLE, J.D. & BALDWIN, R.W. (1986*a*). Immunogenicity of newly established colorectal cell lines. *Br. J. Cancer*, **53**, 37.

DURRANT, L.G., ROBINS, R.A., ARMITAGE, N.C., BROWN, A., BALDWIN, R.W. & HARDCASTLE, J.D. (1986*b*). Association of antigen expression and DNA ploidy in human colorectal tumors. *Cancer Research*, **46**, 3543.

EMBLETON, M.J., GUNN, B., BYERS, V.S. & BALDWIN, R.W. (1981). Antitumour reactions of monoclonal antibody against a human osteogenic sarcoma cell line. *Br. J. Cancer*, **43**, 582.

EMBLETON, M.J., ROWLAND, G.F., SIMMONDS, R.G., JACOBS, E., MARSDEN, C.H. & BALDWIN, R.W. (1983). Selective cytotoxicity against human tumour cells by a vindesine-monoclonal antibody conjugate. *Br. J. Cancer*, **47**, 43.

FARRANDS, P.A., PERKINS, A.C., PIMM, M.V., HARDY, J.D., BALDWIN, R.W. & HARDCASTLE, J.D. (1982). Radioimmunodetection of human colorectal cancer using an antitumour monoclonal antibody. *Lancet*, **ii**, 397.

FARRANDS, P.A., PERKINS, A.C., SULLEY, L. *et al.* (1983). Localisation of human osteosarcoma by anti-tumour monoclonal antibody 791T/36. *J. Bone Joint Surg.*, **65**, 638.

GARNETT, M.C., EMBLETON, M.J., JACOBS, E. & BALDWIN, R.W. (1983). Preparation and properties of a drug-carrier-antibody conjugate showing selective antibody-directed cytotoxicity *in vitro*. *Int. J. Cancer*, **31**, 661.

HOLMES, C.H., HAWKEY, C.J., GUNN, B. & 5 others. (1983). A monoclonal antibody reactive with human hepatocytes. *Liver*, **3**, 295.

PRICE, M.R., CAMPBELL, D.G. & BALDWIN, R.W. (1983). Indentification of an anti-human osteogenic sarcoma monoclonal-antibody-defined antigen on mitogen-stimulated peripheral blood mononuclear cells. *Scand. J. Immunol.*, **18**, 411.